# Differential modulation of the TRAIL receptors and the CD95 receptor in colon carcinoma cell lines

**DOI:** 10.1038/sj.bjc.6601065

**Published:** 2003-07-15

**Authors:** C M M van Geelen, E G E de Vries, T K P Le, R P van Weeghel, S de Jong

**Affiliations:** 1 Department of Medical Oncology, University Hospital of Groningen, PO Box 30. 001, 9700RB Groningen, The Netherlands; 2 IQ Corporation, Groningen, The Netherlands

**Keywords:** TRAIL, TRAIL receptors, CD95, apoptosis, colon carcinoma

## Abstract

Tumour necrosis factor-related apoptosis-inducing ligand (TRAIL) and CD95 ligand (CD95L) are potent inducers of apoptosis in various tumour cell types. Death receptors DR4 and DR5 can induce and decoy receptors DcR1 and DcR2 can inhibit TRAIL-mediated apoptosis. The study aim was to investigate whether anticancer agents can modulate similarly TRAIL-receptor and CD95 membrane expression and TRAIL and CD95L sensitivity.Three colon carcinoma cell lines (Caco-2, Colo320 and SW948) were treated with 5-fluorouracil (5-FU), cisplatin or interferon-*γ*. TRAIL-receptor and CD95 membrane expression was determined flow cytometrically. Sensitivity to TRAIL or CD95L agonistic anti-CD95 antibody was determined with cytotoxicity and apoptosis assays. SW948 showed highest TRAIL sensitivity. The protein synthesis inhibitor cycloheximide decreased FLICE-like inhibitory protein levels in all cell lines, and the TRAIL-resistant cell lines Caco-2 and Colo320 became sensitive for TRAIL. Exposure of the cell lines to 5-FU, cisplatin and interferon-*γ* left TRAIL-receptor membrane expression and TRAIL sensitivity unaffected. CD95 membrane expression and anti-CD95 sensitivity was, however, modulated by the same drugs in all lines. Cisplatin and interferon-*γ* raised CD95 membrane levels 6–8-fold, interferon-*γ* also increased anti-CD95 sensitivity. These results indicate that the CD95 and TRAIL pathways use different mechanisms to respond to various anticancer agents. Induced CD95 membrane upregulation was associated with increased anti-CD95 sensitivity, whereas no upregulation of TRAIL-receptor membrane expression or TRAIL sensitisation could be established. For optimal use of TRAIL-mediated apoptosis for cancer therapy in certain tumours, downregulation of intracellular inhibiting factors may be required.

Colorectal cancer is the third most frequent cause of cancer-related death (Ilyas *et al*, 1999). Since resistance to chemotherapeutic drugs remains a major problem in the treatment of this disease, the use of death receptor ligands as potential therapeutic agents is of great interest. The death receptor ligands tumour necrosis factor-*α* (TNF-*α*), CD95 ligand (CD95L) and TNF-related apoptosis-inducing ligand (TRAIL) induce apoptosis in a variety of cells after ligation with their receptors. TRAIL shows great homology with CD95L. Certain cellular apoptotic proteins are involved in the cellular pathways of both ligands, suggesting that these pathways converge to some extent. Systemic use of CD95 is impossible due to severe toxic side effects. TRAIL on the other hand is a potent inducer of apoptosis in cancer cells *in vitro* (Wiley *et al*, 1995; Pitti *et al*, 1996) and in its native biological form, it exhibits antitumour activity in several xenograft studies *in vivo* without serious toxicity (Ashkenazi *et al*, 1999; Gliniak and Le, 1999; Walczak *et al*, 1999).

TRAIL can bind to four membrane receptors. Two of these receptors, DR4 (Pan *et al*, 1997b) and DR5 (Chaudhary *et al*, 1997; MacFarlane *et al*, 1997; Pan *et al*, 1997a; Schneider *et al*, 1997; Sheridan *et al*, 1997), are receptors that contain a cytoplasmic death domain through which TRAIL can mediate an apoptotic signal to the cell. Two other receptors, DcR1 and DcR2 can also bind TRAIL, but lack an intact death domain (Degli-Eposti *et al*, 1997; MacFarlane *et al*, 1997; Marsters *et al*, 1997; Pan *et al*, 1997a, 1998; Sheridan *et al*, 1997). Consequently, DcR1 and DcR2 are thought to act as decoy receptors, protecting the cell from apoptosis (Pan *et al*, 1997a; Sheridan *et al*, 1997).

TRAIL binds as a homotrimer to DR4 and DR5, which upon binding will trimerise and form a death-inducing signalling complex. Initiator caspases, such as caspase 8, will be activated by autocleavage and activate downstream effector caspases such as caspase 3, and finally the cell will go into apoptosis (Ashkenazi and Dixit, 1998).

Treatment with cytokines and chemotherapeutic drugs can upregulate protein levels (Nimmanapalli *et al*, 2001) and mRNA of DR4 and DR5 in p53-dependent or independent ways (Sheikh *et al*, 1998; Wu *et al*, 2000) and augment TRAIL sensitivity (Keane *et al*, 1999; Nimmanapalli *et al*, 2001). However, almost no studies have looked into whether anticancer drugs can change TRAIL-receptor membrane expression, while this effect has been described for CD95 (Timmer *et al*, 2002). Furthermore, it is not known whether this change in receptor expression levels at the cell surface or expression levels of pro- and antiapoptotic proteins are the key determinants for sensitivity to TRAIL or CD95L. To address these questions, we studied membrane expression levels of TRAIL receptors in relation to TRAIL sensitivity in a panel of three colon carcinoma cell lines. Furthermore, we analysed modulation of TRAIL-receptor membrane expression and TRAIL sensitivity by anticancer drugs. We compared this outcome with the effect these drugs exhibit on the CD95-receptor and sensitivity to agonistic anti-CD95 in the three colon carcinoma cell lines. In addition, we analysed how downregulation of intracellular components of the apoptotic pathways was related to TRAIL sensitivity and sensitivity to anti-CD95. From the results we can conclude that the CD95 pathway and the TRAIL pathway respond differently to various anticancer agents.

## MATERIALS AND METHODS

### Reagents

RPMI 1640 medium was obtained from Life Technologies (Breda, The Netherlands) and foetal calf serum (FCS) from Bodinco BV (Alkmaar, The Netherlands). Cisplatin and 5-fluorouracil (5-FU) were purchased from Pharmachemie BV (Haarlem, The Netherlands), interferon-*γ* was obtained from Roche Diagnostics (Mannheim, Germany) and TNF-*α* was kindly provided by Boehringer Ingelheim (Ingelheim am Rhein, Germany). 3-(4,5-Dimethylthiazol-2-yl) 2,5-diphenyltetrazolium bromide (MTT) and cycloheximide were purchased from Sigma-Aldrich (Zwijndrecht, The Netherlands). Anti-CD95 7C11 was obtained from Immunotech (Marseille, France). Recombinant human TRAIL (rhTRAIL) was produced noncommercially in cooperation with IQ-Corporation (Groningen, The Netherlands) following a protocol described elsewhere (Ashkenazi *et al*, 1999). The TRAIL-receptor antibodies used for flow cytometry were obtained from Immunex Corporation (Seattle, WA, USA).

### Cell lines

For this study, the colon carcinoma cell lines Caco-2 (Fogh *et al*, 1977), Colo320 (Quinn *et al*, 1979) and SW948 (Leibovitz *et al*, 1976) were used. Caco-2 and Colo320 were cultured in RPMI 1640 medium supplemented with, respectively, 13 and 10% foetal calf serum at 37°C in a humidified atmosphere with 5% CO_2_. SW948 was cultured in Leibovitz L15-RPMI 1640 (1 : 1) enriched with 10% FCS, 0.05 M pyruvate, 0.1 M glutamine and 0.025% *β*-mercaptoethanol at 37°C in a humidified atmosphere with 5% CO_2_. Caco-2 and SW948 were harvested by treatment with protease XXIV for 5–10 min at 37°C.

### Cytotoxicity assay

The microculture tetrazolium assay was used to determine cytotoxicity. In a total volume of 200 *μ*l, 3750 cells for Caco-2, 1000 cells for Colo320 and 3750 cells for SW948 were incubated. Treatment consisted of continuous incubation with various concentrations of 5-FU or interferon-*γ* with or without anti-CD95 or rhTRAIL. Treatment with cisplatin consisted of 24 h preincubation with cisplatin alone before adding anti-CD95 or rhTRAIL. After an incubation period of 96 h, 20 *μ*l of MTT-solution (5 mg ml^−1^ phosphate-buffered saline (PBS: 6.4 mM Na_2_HPO_4_; 1.5 mM KH_2_PO_4_; 0.14 mM NaCl; 2.7 mM KCl; pH=7.2)) was added for 3.75 h. Subsequently, plates were centrifuged and the supernatant aspirated. After dissolving the formazan crystals by adding dimethyl sulphoxide (Merck, Amsterdam, The Netherlands), plates were read immediately at 520 nm using a microtitre well spectrometer (Bio-Rad microplate reader, Bio-Rad laboratories B.V., Veenendaal, The Netherlands). Controls consisted of media without cells. Cell survival was defined as the growth of treated cells compared to untreated cells. IC_50_ is the concentration of drug inhibiting survival by 50%. Mean cytotoxicity was determined in three independent experiments, each performed in quadruplicate.

### Sodium dodecyl sulphate (SDS)–polyacrylamide gel electrophoresis (PAGE) and Western blotting

Cells were washed twice in cold PBS. Then they were lysed in SDS sample buffer (10% 2-*β*-mercaptoethanol, 4% SDS, 0.5 M Tris-HCl, pH 6.8 and 20% glycerol, 0.002% bromophenol blue) and boiled in a waterbath for 5 min. Proteins were separated on a polyacrylamide SDS containing gel and transferred to a polyvinylidene difluoride membrane (PVDF membrane) (Millipore BV, Etten-Leur, The Netherlands). Nonspecific binding sites were blocked with 5% skimmed milk (Fluka) in TBS-Tween (TBS with 0.05% TWEEN (Sigma, Aldrich (Zwyndrecht, The Netherlands))) for at least 1 h at room temperature. After blocking, the membranes were incubated with specific antibodies for 1 h at room temperature. To detect CD95-associated death domain (FADD), X-linked inhibitor of apoptosis protein (XIAP), FLICE-like inhibitory protein (FLIP), caspase 3, caspase 9, caspase 8, poly (ADP-ribose)-polymerase (PARP), DR4, DR5, DcR2, CD95, p53 and Fas-associated phosphatase-1 (FAP-1), CD95L and TRAIL, the following antibodies were used: mouse-anti-FADD, mouse-anti-XIAP and mouse-anti-CD95L from Transduction Laboratories (Lexington, KY, USA), rabbit-anti-caspase 3 and rabbit-anti-caspase 9 from Pharmingen (Becton Dickinson, Erebodegem-Aalst, Belgium), rabbit-anti-PARP from Roche Diagnostics (Mannheim, Germany), goat-anti-DR4, goat-anti-CD95, mouse-anti-p53, goat-anti-TRAIL(K18) and goat-anti-FAP-1 from Santa Cruz Biotechnology (Santa Cruz, CA, USA) rabbit-anti-DR5 and rabbit-anti-DcR2 from Oncogene Research Products (Calbiochem-Novabiochem, Germany). Mouse anti-caspase 8 was purchased from Cell Signaling Technology (Leusden, The Netherlands). Mouse-anti-FLIP NF6 was kindly provided by M Peter (Chicago, IL, USA). Mouse-antiactin was obtained from ICN Biomedicals (Zoetermeer, The Netherlands).

The secondary antibodies were labelled with horseradish peroxidase (all from DAKO, Glostrup, Denmark), and chemiluminesence was detected using the ECL-chemiluminescence kit or with the Lumi-Light Plus Western blotting kit (Roche Diagnostics, Mannheim, Germany). Western blot analyses have been performed at least three times. To study caspase 3 activation and PARP cleavage, cells were treated with rhTRAIL, anti-CD95, cycloheximide, or combinations of cycloheximide with rhTRAIL or anti-CD95 for 24 h. Concentrations of 5 *μ*g ml^−1^ cycloheximide, 0.1 *μ*g ml^−1^ rhTRAIL and 0.05 *μ*g ml^−1^ anti-CD95 were used, unless indicated otherwise. Cells were pretreated with cycloheximide for 1 h before adding rhTRAIL and anti-CD95. To analyse the effect of cycloheximide on the protein level of FLIP-L, the cells were pretreated 1 h with cycloheximide before TRAIL or anti-CD95 was added. For Caco-2 and Colo320 cell lines, the incubation was 24 h, for the TRAIL-sensitive cell line SW948 the incubation time was 5 h for TRAIL and 24 h for anti-CD95. The Bradford (1976) assay was used to determine protein concentrations in all samples. In all experiments, samples containing 20 *μ*g lysate were used, and all membranes were stained with Ponceau S to check for equal protein loading. Protein expression levels were standardised to actin expression and densitometrically analysed with Diversity One (Amersham Biosciences Europe (Roosendaal, The Netherlands) software.

### Flow cytometry

Analysis of CD95 and TRAIL-receptor membrane expression was performed using a flow cytometer (Epics Elite, Coulter-Electronics, Hialeah, FL, USA). Adherent cells were harvested by treatment with protease (0.01 or 0.005%) for 5 min at 37°C and washed twice in PBS at 4°C. Appropriate concentrations of antibodies were added to the cells in 50 *μ*l PBS containing 2% FCS and 0.1% sodium azide. Cells were incubated for 30 min on ice. Cells were washed twice with cold PBS containing 2% FCS and 0.1% sodium azide at 4°C and incubated with FITC-conjugated rabbit-anti-mouse (DAKO, Glostrup, Denmark) for 30 min on ice. After washing, a minimum of 5000 cells were analysed by flow cytometry. For the TRAIL receptors, the mouse IgG1 and mouse IgG2a antibodies (DAKO) were used as isotype controls.

PE-conjugated anti-CD95 antibody and a PE-conjugated mouse IgG1*κ* control were obtained from Pharmingen. Antibodies against the different TRAIL receptors were a gift of Immunex (Seattle, WA, USA). For TRAIL-receptor 1, huTRAILR1-M271, for TRAIL-receptor 2, huTRAILR2-M413, for TRAIL-receptor 3, huTRAILR3-M430, and for TRAIL-receptor 4, huTRAILR4-M444 has been used. Membrane receptor expression is shown as mean fluorescent intensity (MFI) of all analysed cells. Membrane expression was observed as an increase in fluorescence intensitiy for the whole analysed cell population. All experiments are performed at least three times.

### Reverse transcription–polymerase chain reaction (RT–PCR)

Total RNA was isolated by guanine thiocyanate–phenol–chloroform extraction and treated with DNAse for 30 min at 37°C to remove genomic DNA contamination. cDNA was synthesised from 5 *μ*g RNA as described by the manufacturer's protocol (Life Technologies, Breda, The Netherlands) using oligo (dT) primers and MMLV transcriptase (Life Technologies, Breda, The Netherlands). The sequences for the relevant primers were as described elsewhere (Griffith *et al*, 1998; Rieger *et al*, 1999). Annealing temperatures for DR4, DR5, DcR1, DcR2 and TRAIL were 61, 61, 61, 60 and 62°C, respectively. The DNA fragments were amplified for 30 cycles using a thermal sequencer (Perkin Elmer Benelux (Groningen, The Netherlands)). The program consisted of one cycle of 96°C for 1 min, 30 cycles of 96°C for 45 s, the specific annealing temperature for 45 s, 72°C for 45 s and one cycle of 72°C for 10 min. The PCR-amplified products of DR4, DR5, DcR1, DcR2 and TRAIL were, respectively, 506, 502, 612, 464, and 413 bp in size. PCR products were run on a 2% agarose gel in 1 × Tris-borate EDTA (TBE) buffer and were visualised under UV light. PCR products were checked for the right size with restriction enzymes. The housekeeping gene GAPDH was used as a control.

### Apoptosis assay

From each cell line, 1.0 × 10^4^ cells were seeded in 96-well plates with or without interferon-*γ* (10 or 100 U ml^−1^). After 24–30 h incubation at 37°C, cycloheximide (5 *μ*g ml^−1^) was added. After 1 h incubation with cycloheximide, rhTRAIL (0.1 or 1.0 *μ*g ml^−1^), anti-CD95 (0.05 *μ*g ml^−1^) or TNF-*α* (0.1 or 1.0 *μ*g ml^−1^) was added and the cells were incubated for 24 h at 37°C. Apoptosis was identified by staining nuclear chromatin with acridine orange, identifying morphological changes by fluorescence microscopy. Control cells were seeded with medium, cycloheximide or interferon-*γ* alone. Apoptosis experiments were performed at least three times.

### Caspase 3 activation assay

Activity of caspase 3 in Caco-2, Colo320 and SW948 cells treated with 5 *μ*g ml^−1^ cycloheximide, 0.05 *μ*g ml^−1^ anti-CD95, 0.1 *μ*g ml^−1^ rhTRAIL or combinations of cycloheximide with anti-CD95 or rhTRAIL was determined. Cells were exposed to these compounds for 24 h, except for SW948. For this cell line anti-CD95 incubations were 24 h, whereas rhTRAIL incubations were 3 h. Activity of caspase 3 was assayed according to the manufacturer's instructions using the fluorescence peptide substrate Ac-DEVD-AFC (Biomol tebu-bio, Heerhugowaard, The Netherlands). Fluorescence from free 7-amino-4-trifluoromethyl coumarin (AFC) was monitored in a FL600 Fluorimeter Bio-tek plate reader (Beun de Ronde, Abcoude, The Netherlands) using 380 nm excitation and 508 nm emission wavelengths. Relative caspase 3 activity was expressed as the ratio of treated to untreated cells. Experiments are performed for at least three times. Values are mean±s.d.'s.

### Statistics

Statistical an analysis was performed using the Student's *t*-test. *P*-values ⩽0.05 were considered to be significant.

## RESULTS

### The three colon carcinoma cell lines differ in TRAIL sensitivity

**Figure 1:**
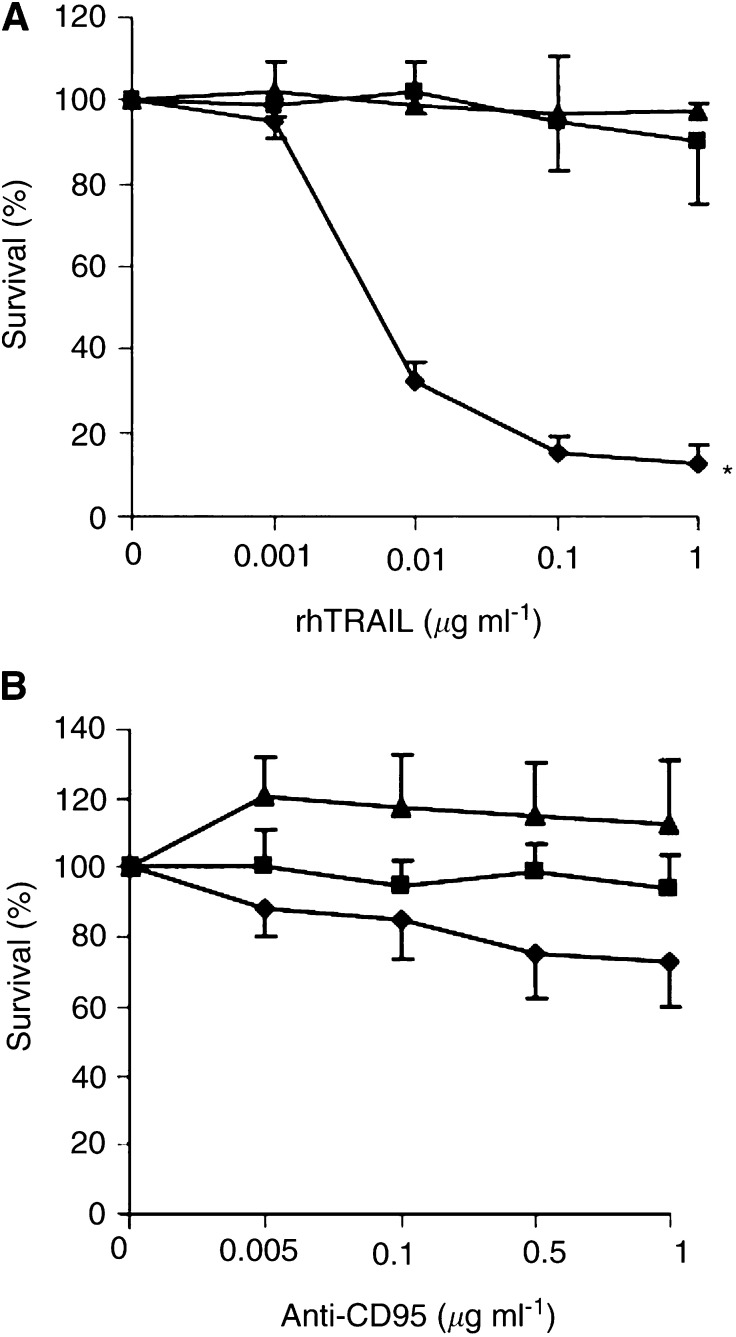
Survival (%) of Caco-2 (closed square), Colo320 (closed triangle) and SW948 (closed diamond), after continuous incubation with (**A**) rhTRAIL and (**B**) anti-CD95 as measured by cytotoxicity assays. Values are mean±s.d. of at least three independent experiments. ^*^: SW948 differs from Caco-2 and Colo320 at 0.01–1.0 *μ*g ml^−1^ rhTRAIL (*P*<0.05).

Sensitivity of the three colon carcinoma cell lines to the cytotoxic activity of 0.001–1.0 *μ*g ml^−1^ rhTRAIL for 96 h was determined using the cytotoxicity assay (Figure 1A

).

**Figure 2:**
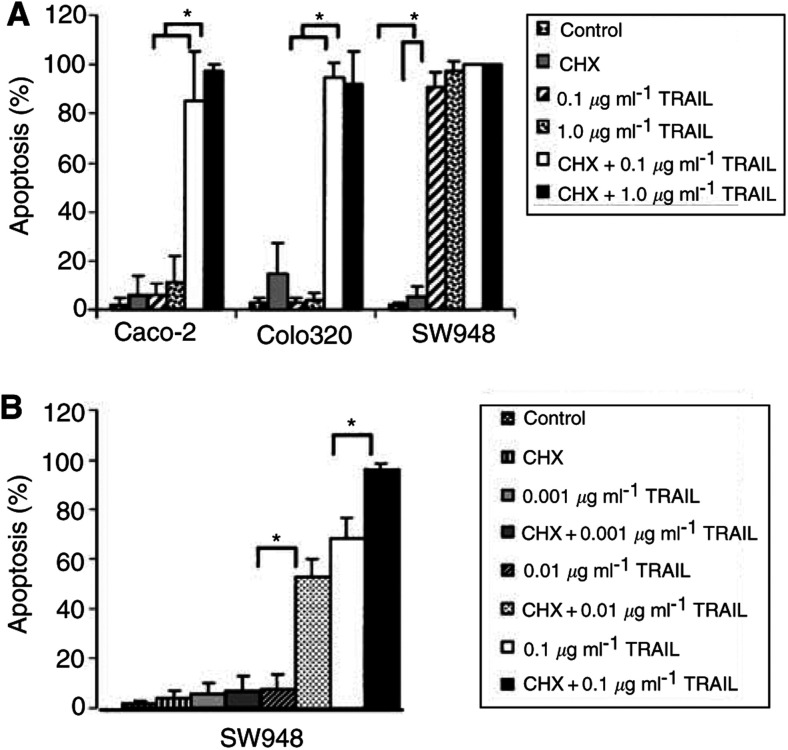
(**A**) Apoptosis assay for Caco-2, Colo320 and SW948. Cells were incubated for 24 h in medium; with 5 *μ*g ml^−1^ cycloheximide (CHX); with 0.1 or 1.0 *μ*g ml^−1^ rhTRAIL; with the combination cycloheximide and 0.1 or 1.0 *μ*g ml^−1^ rhTRAIL. Values are mean±s.d. of at least three independent experiments. ^*^: the percentage apoptotic Caco-2 or Colo320 cells in the presence of cycloheximide plus rhTRAIL differs from the percentage apoptotic cells in the presence of rhTRAIL alone (*P*<0.05). The percentage of apoptotic SW948 cells in the presence of rhTRAIL differs from the percentage of apoptotic cells in the control cells (*P*<0.05). (**B**) Apoptosis assay for SW948. Cells were incubated for 3 h in medium; with 5 *μ*g ml^−1^ cycloheximide; with 0.1, 0.01 or 0.001 *μ*g ml^−1^ rhTRAIL; with the combination cycloheximide and 0.1, 0.01 or 0.001 *μ*g ml^−1^ rhTRAIL. Values are mean±s.d. for at least three independent experiments. ^*^: the percentage of apoptotic SW948 cells in the presence of cycloheximide plus rhTRAIL differs from the percentage of apoptotic cells in the presence of rhTRAIL alone, (*P*<0.05).

**Figure 3:**
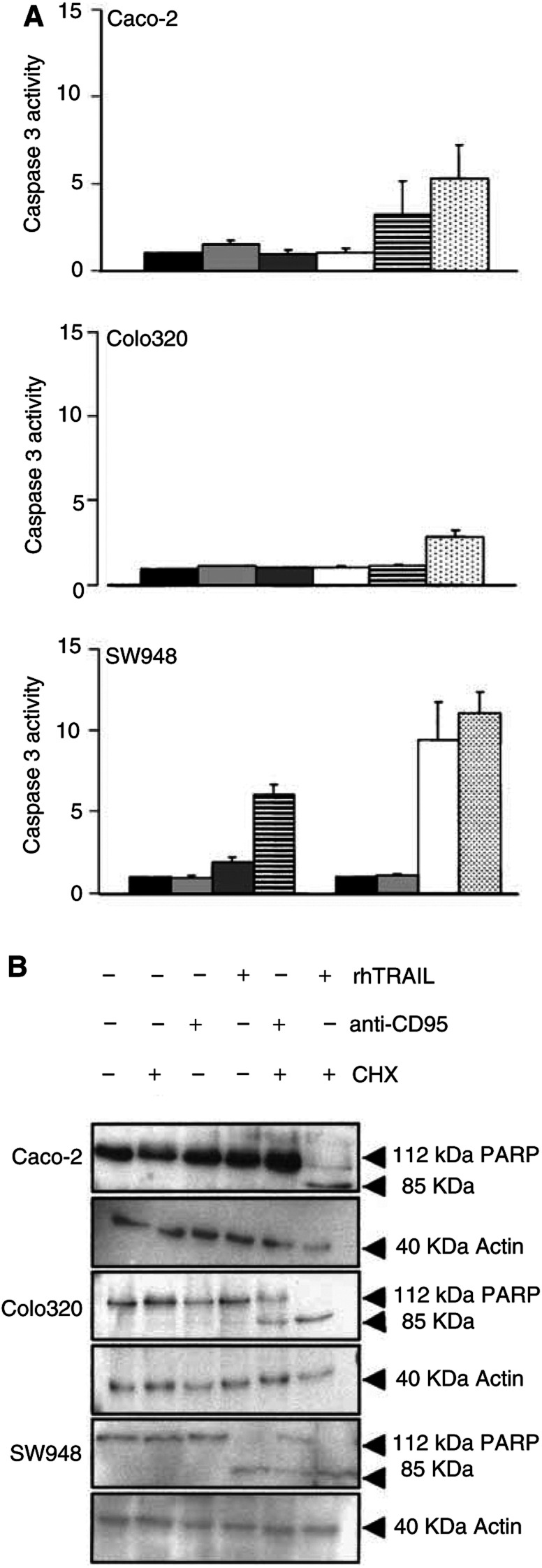
(**A**) Caspase 3 activity assay for Caco-2, Colo320 and SW948. Cells were incubated as control in medium (black bars), cycloheximide (light grey bars), anti-CD95 (dark grey bars), rhTRAIL (white bars) or combinations of cycloheximide with anti-CD95 (striped bars) or cycloheximide with rhTRAIL (spotted bars) as described in Materials and Methods. Caspase 3 activity is expressed as the ratio of treated to untreated cells. Experiments are performed for at least three times, values are mean±s.d.'s. (**B**) Western blot analysis of PARP cleavage in Caco-2, Colo320 and SW948 after treatment with cycloheximide, rhTRAIL, anti-CD95, rhTRAIL and cycloheximide or anti-CD95 and cycloheximide. If cells are apoptotic, full-length PARP (112 kDa) is cleaved into an 85 kDa fragment. One representative of at least three different experiments is shown. Actin (40 kDa) is shown as a loading control.

SW948 is very sensitive to rhTRAIL with an IC_50_ of 0.007 *μ*g ml^−1^. Caco-2 and Colo320 are resistant to rhTRAIL even at the highest concentration of 1 *μ*g ml^−1^ (Figure 1A). The cytotoxic effect of rhTRAIL is related to its ability to trigger apoptotic cell death. As demonstrated in Figure 2

, an increasing concentration of rhTRAIL resulted in increasing percentage of apoptotic cells in SW948. Apoptosis was also confirmed with cleavage of caspase 3 and cleavage of the early apoptosis marker PARP (Figure 3

).

### Determining the functionality of the TRAIL-mediated apoptosis pathway

**Figure 4:**
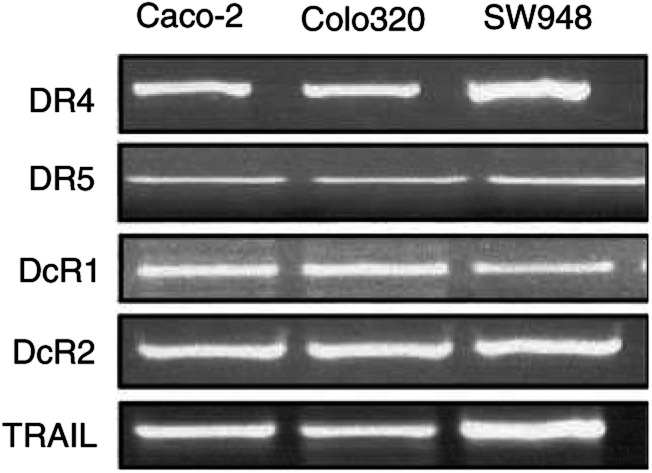
Reverse transcription–PCR of TRAIL and TRAIL receptors in Caco-2, Colo320 and SW948, as described in the Materials and Methods. One representative of at least three different experiments is shown.

**Figure 5:**
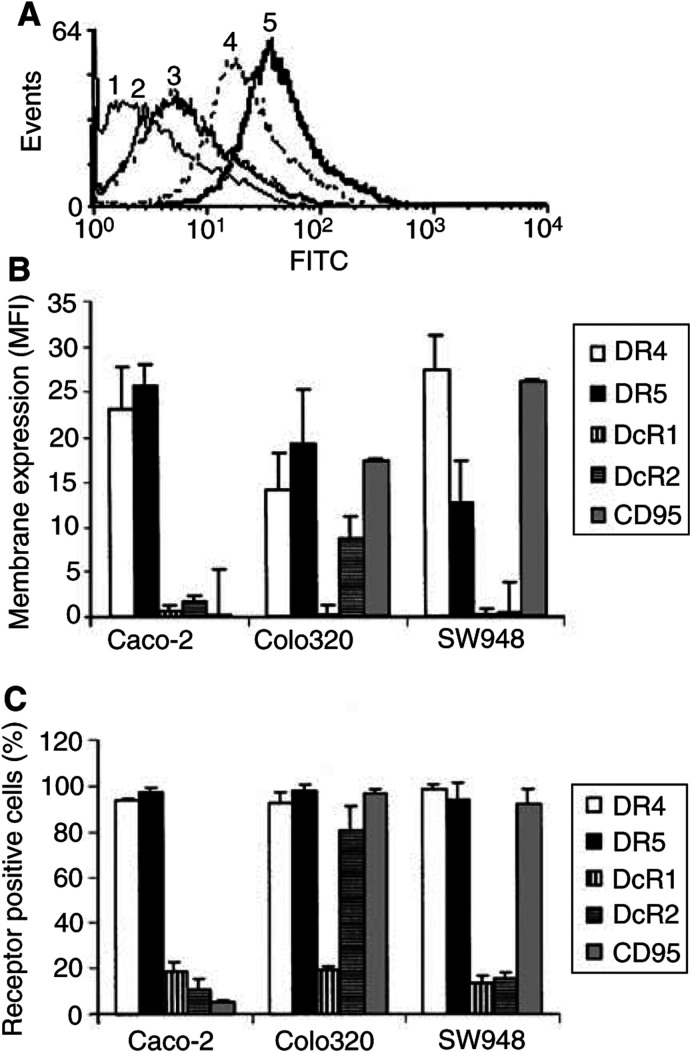
Basic membrane expression of the TRAIL receptors DR4, DR5, DcR1 and DcR2 and the CD95 receptor in Caco-2, Colo320 and SW948 as determined by flow cytometry. (**A**) A representative example of TRAIL-receptor membrane expression in SW948. Receptor expression was detected as an increased fluorescence intensity of the whole cell population and resulted in a peak shift to the right (1=control; 2=DcR1; 3=DcR2; 4=DR5; 5=DR4). (**B**) TRAIL and CD95 receptor membrane expression of Caco-2, Colo320 and SW948 expressed as MFI. Values are mean±s.d. of at least three independent experiments. (**C**) The percentages CD95 and TRAIL-receptor positive cells analysed with flow cytometry. Values are mean±s.d.'s of three different experiments.

To further analyse the functionality of the TRAIL-mediated apoptosis pathway in these three cell lines, TRAIL-receptor expression on the mRNA, protein and membrane level was determined. Since mRNAs of all TRAIL receptors and TRAIL could be detected in the three cell lines using RT–PCR (Figure 4

), expression of the receptors on the cell surface was studied by flow cytometry. As demonstrated in Figure 5

, SW948 expressed more DR4 than DR5 on the membrane, whereas Caco-2 and Colo320 expressed similar levels of the death receptors on the surface. Caco-2 and SW948 had very low or no expression of DcR1 or DcR2 on their surface, while Colo320 showed a low DcR2 expression.

**Figure 6:**
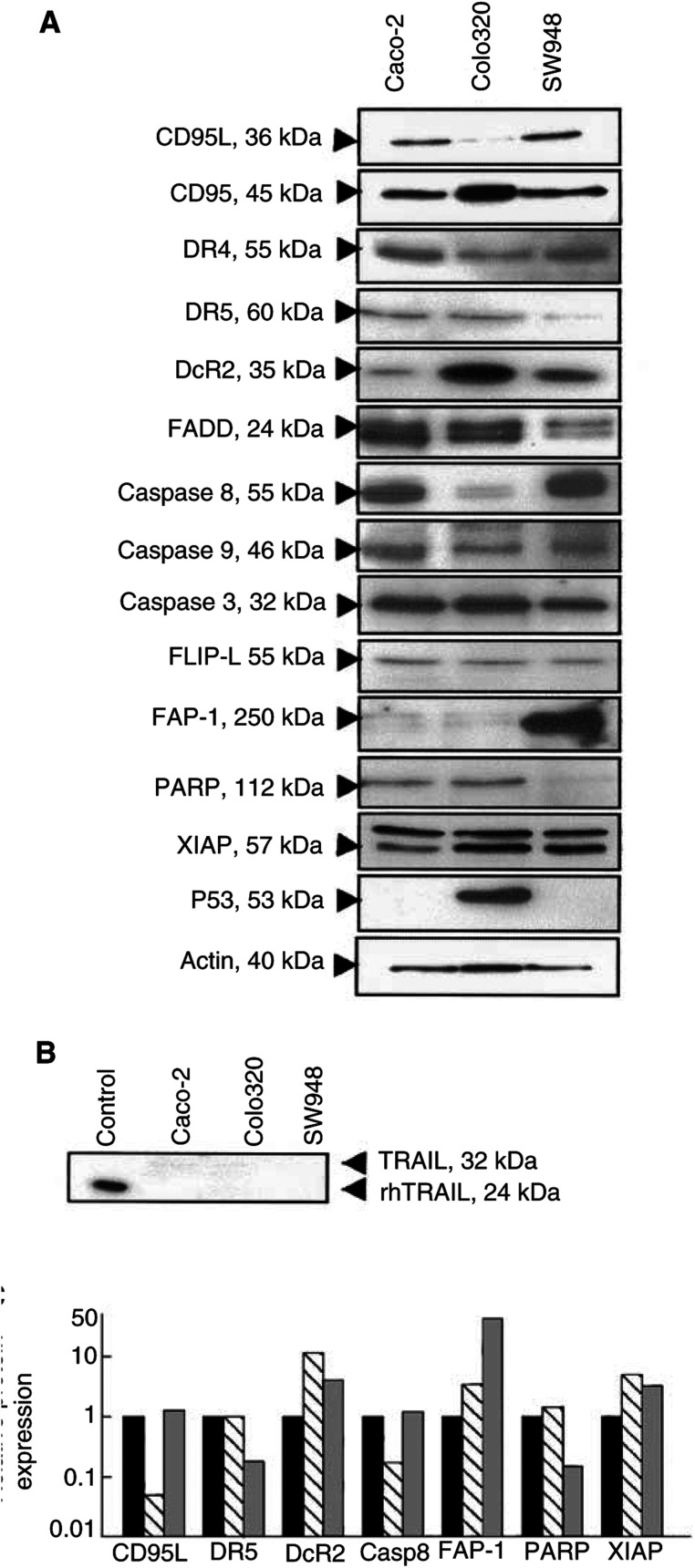
(**A**) Western blot analysis of basic expression levels of several proteins involved in TRAIL- and CD95-mediated apoptosis in Caco-2, Colo320 and SW948 cells. One representative of at least three different experiments is shown. Bands of interest are indicated with arrows. (**B**) Western blot analysis of TRAIL protein expression in Caco-2, Colo320 and SW948. Recombinant human TRAIL (50 ng) was loaded as a positive control. (**C**) Relative optical density of Caco-2 (black bars), Colo320 (striped bars) and SW948 (grey bars) protein expression of CD95L, DR5, DcR2, caspase 8 (casp-8), FAP-1, PARP and XIAP. Protein expression was corrected to actin expression and is expressed in relation to Caco-2 expression.

Total protein expression of the TRAIL receptors in these cell lines was studied by Western blot analysis (Figure 6

). No difference in protein expression was found for DR4. SW948 expressed less DR5 than Caco-2 and Colo320. Colo320, the cell line with the highest DcR2 expression on the surface, showed a higher DcR2 total protein expression. Although TRAIL mRNA expression was found (Figure 4), no protein expression of TRAIL could be detected in any of the cell lines (Figure 6B). To further characterise these cell lines, expression levels of several important proteins involved in CD95 and TRAIL-receptor-dependent apoptosis were measured by Western blotting. A few important differences between the cell lines were found and are shown in Figure 6. Colo320 had a lower expression of caspase 8 and CD95L, but a higher expression of XIAP. SW948 had a lower PARP expression, but a higher expression of the inhibitor FAP-1. Caco-2 and SW948 did not express p53. No clear differences in protein expression levels were found for FLIP, FADD, DR5, caspase 3, caspase 9 and CD95.

**Figure 7:**
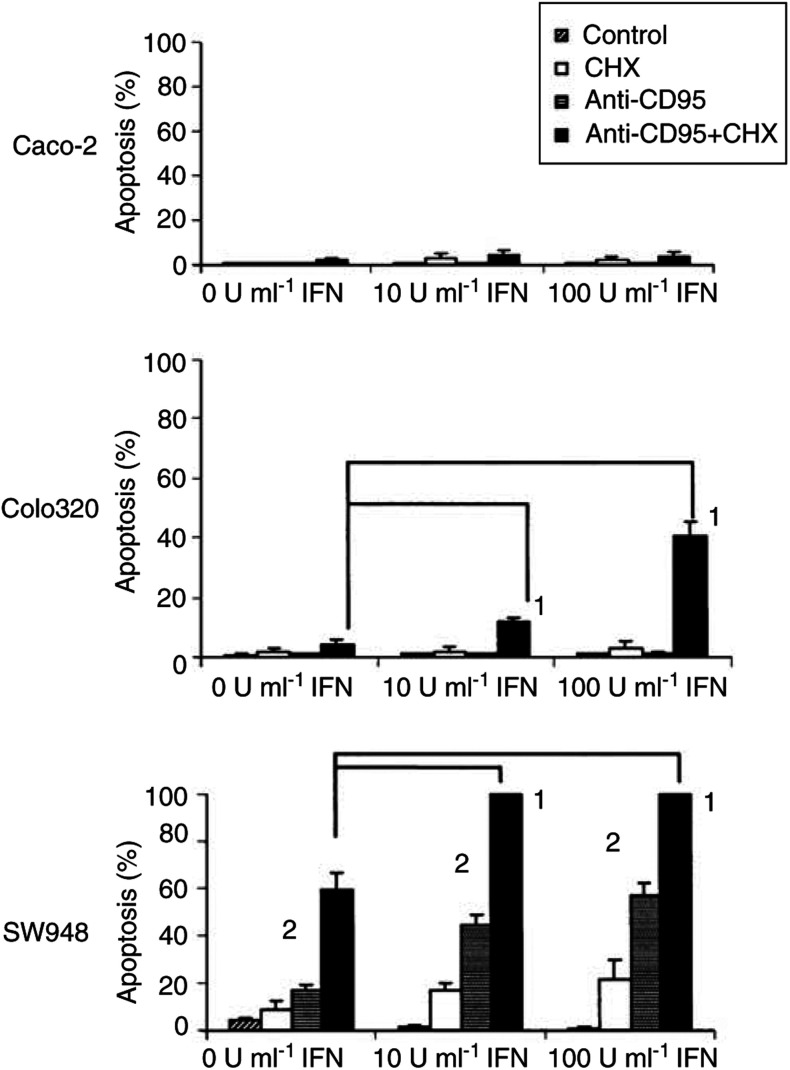
Anti-CD95-mediated apoptosis in Caco-2, Colo320 and SW948 with or without 24 h incubation with interferon-*γ* (IFN). Values are mean +s.d. of at least three independent experiments. **1**: The percentages of apoptotic cells treated with 10 or 1000 U ml^−1^ interferon-*γ* in the presence of cycloheximide plus anti-CD95 differ from the percentage of apoptotic cells in the presence of cycloheximide plus anti-CD95 in the untreated cells (*P*<0.05). **2**: At the same concentration of interferon-*γ*, the percentage of apoptosis in cells treated with anti-CD95 differs from the percentage of apoptosis in cells treated with anti-CD95 and cycloheximide (*P*<0.05).

In the presence of the protein synthesis inhibitor cycloheximide, the TRAIL-resistant cell lines Caco-2 and Colo320 became sensitive to rhTRAIL. Even at the lower concentration of 0.1 *μ*g ml^−1^ rhTRAIL, about 90% of the Caco-2 and Colo320 cells went into apoptosis, and these levels are comparable with the level of apoptosis seen in SW948 without cycloheximide. The fact that Caco-2 and Colo320 become sensitive to rhTRAIL in the presence of cycloheximide indicates that the TRAIL-mediated apoptotic pathway is functional in these cell lines (Figures 2 and 3). If lower rhTRAIL concentrations and shorter incubation time were used, cycloheximide also sensitised SW948 to rhTRAIL (Figure 2B). This indicates that cycloheximide sensitises all three cell lines and that it rendered the already sensitive cell line SW948 even more sensitive. Cycloheximide also sensitised these cell lines for anti-CD95 and TNF-*α* (Figures 7

and 8

**Figure 8:**
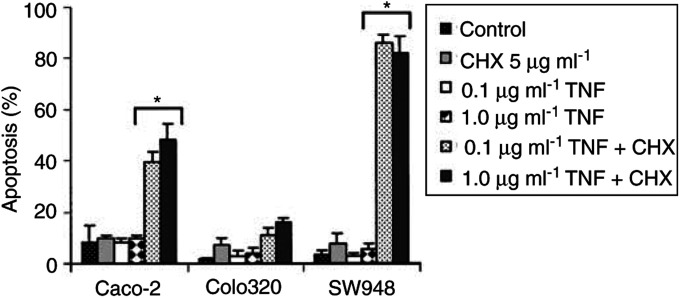
TNF-*α*-mediated apoptosis in Caco-2, Colo320 and SW948. Cells were incubated for 24 h in medium; with 5 *μ*g ml^−1^ cycloheximide (CHX); with 0.1 or 1.0 *μ*g ml^−1^ TNF-*α* (TNF); with 0.1 or 1.0 *μ*g ml^−1^ TNF-*α* in combination with 5 *μ*g ml^−1^ cycloheximide. ^*^: the percentages of apoptotic cells treated with TNF-*α* and cycloheximide differ from the percentages of apoptotic cells with TNF-*α* alone (*P*<0.05).

). Caco-2 is not sensitive to anti-CD95 with or without cycloheximide, but in the presence of cycloheximide this cell line could be sensitised to TNF-*α*. Colo320 can only be sensitised by cycloheximide to anti-CD95 in combination with interferon-*γ*. The presence of cycloheximide only slightly increases the percentage of TNF-*α*-mediated apoptosis. SW948 in contrast, can be sensitised to TNF-*α* as well as to anti-CD95 by cycloheximide. Western blot showed that cycloheximide treatment resulted in a decrease in FLIP-L protein level in all cell lines with only an almost undetectable level in Colo320 (Figure 9

**Figure 9:**
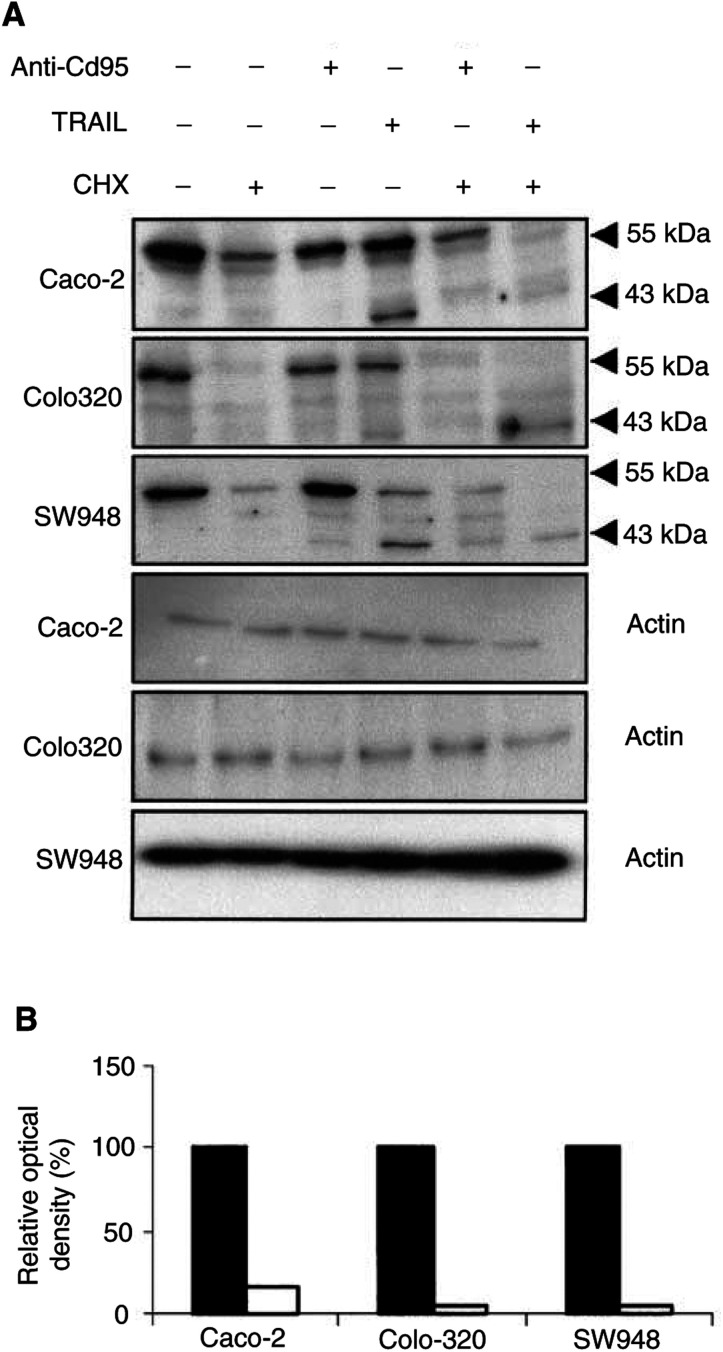
(**A**) Western blot analysis determined that cycloheximide (CHX) decreased FLIP protein levels in Caco-2, Colo320 and SW948. After exposure to rhTRAIL or anti-CD95, full-length FLIP-L (55 kDa) is cleaved into an intermediate product of 43 kDa. One representative of at least three different experiments is shown. Actin (40 kDa) is shown as loading control. (**B**) Relative protein expression (%) of FLIP in controls (black bars) and cycloheximide-treated (white bars) cells. FLIP protein expression is corrected for actin expression. Protein expression of cells incubated with cycloheximide is presented in relation to FLIP expression in the control cells.

). Treatment with rhTRAIL alone resulted in FLIP-L cleavage into an intermediate product of 43 kDa in all cell lines. In the rhTRAIL-sensitive SW948 cell line, most FLIP-L was present as the cleaved intermediate product, which is in agreement with the observed caspase 3 activation and PARP cleavage (see Figure 3). Cycloheximide in combination with rhTRAIL resulted in a complete loss of the full-length FLIP-L and low levels of the intermediate product. Incubation with cycloheximide had no effect on Bax, Bcl2 and Bcl-X_L_ protein levels in these cell lines (data not shown).

### Modulation of TRAIL-receptor membrane expression and TRAIL sensitivity

**Figure 10:**
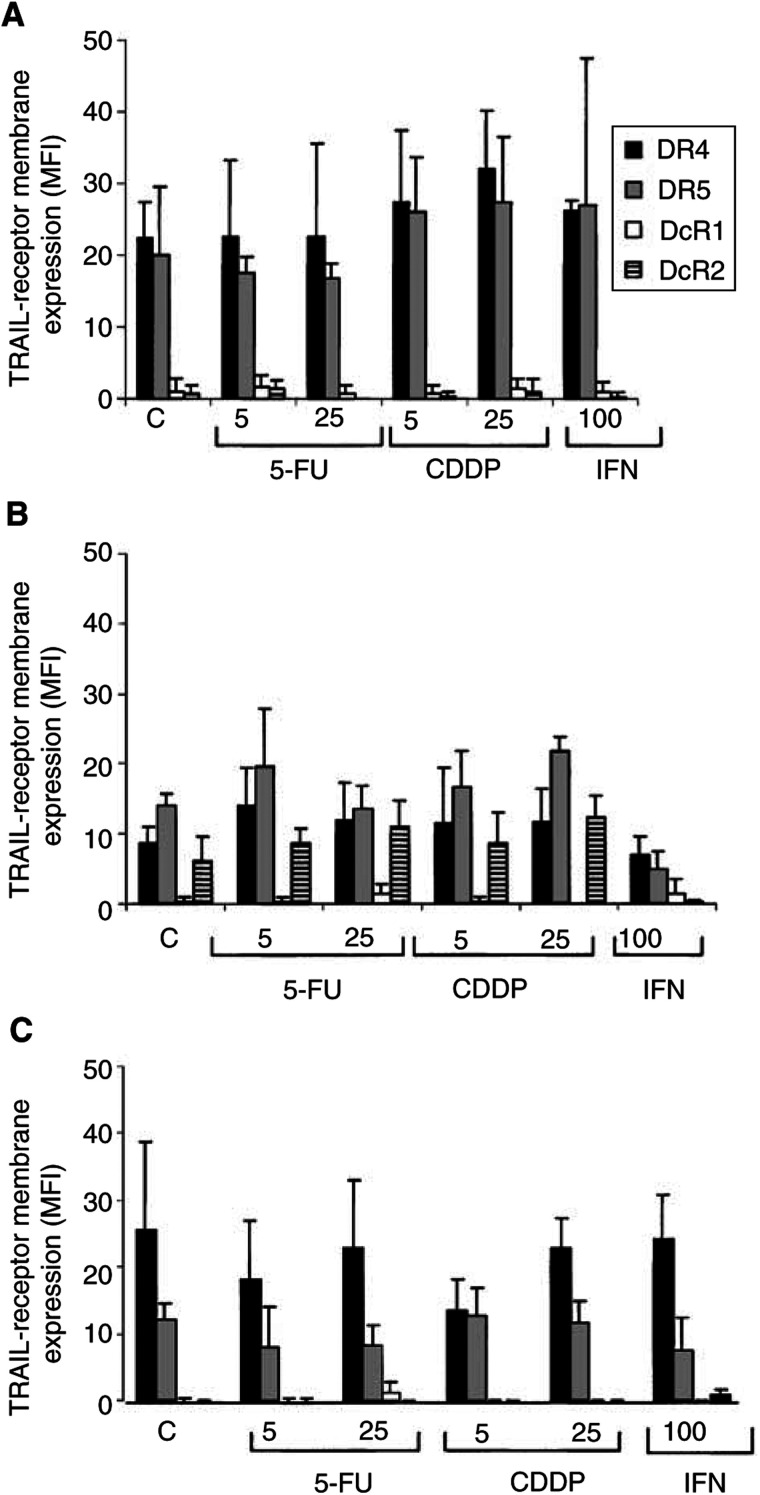
TRAIL-receptor membrane expression in (**A**) Caco-2, (**B**) Colo320 and (**C**) SW948 in control cells, after 24 h exposure to 5 or 25 *μ*M 5-FU, 5 or 25 *μ*M cisplatin (CDDP) or 100 U ml^−1^ interferon-*γ* (IFN). Membrane expression is determined by flow cytometry and expressed as MFI. Exposure to 5-FU, cisplatin or interferon-*γ* did not change the percentage of receptor positive cells. Values are mean±s.d. of at least three independent experiments.

To analyse whether chemotherapeutic drugs can modulate TRAIL-receptor membrane expression, the three cell lines were treated with cisplatin or interferon-*γ*. Cells were incubated for 24 h with 5 and 25 *μ*M 5-FU, 5 and 25 *μ*M cisplatin and 10 or 100 U ml^−1^ interferon-*γ*, and the TRAIL-receptor membrane expression was determined by flow cytometry. Treatment with 5-FU or interferon-*γ* did not result in a clear upregulation of the TRAIL-receptor membrane expression in any of the cell lines (Figure 10

). Interferon-*γ* even decreased membrane receptor expression of Caco-2. Treatment with cisplatin showed a small increase of DR4 and DR5 membrane expression in Caco-2 and Colo320 (Figure 10).

**Figure 13:**
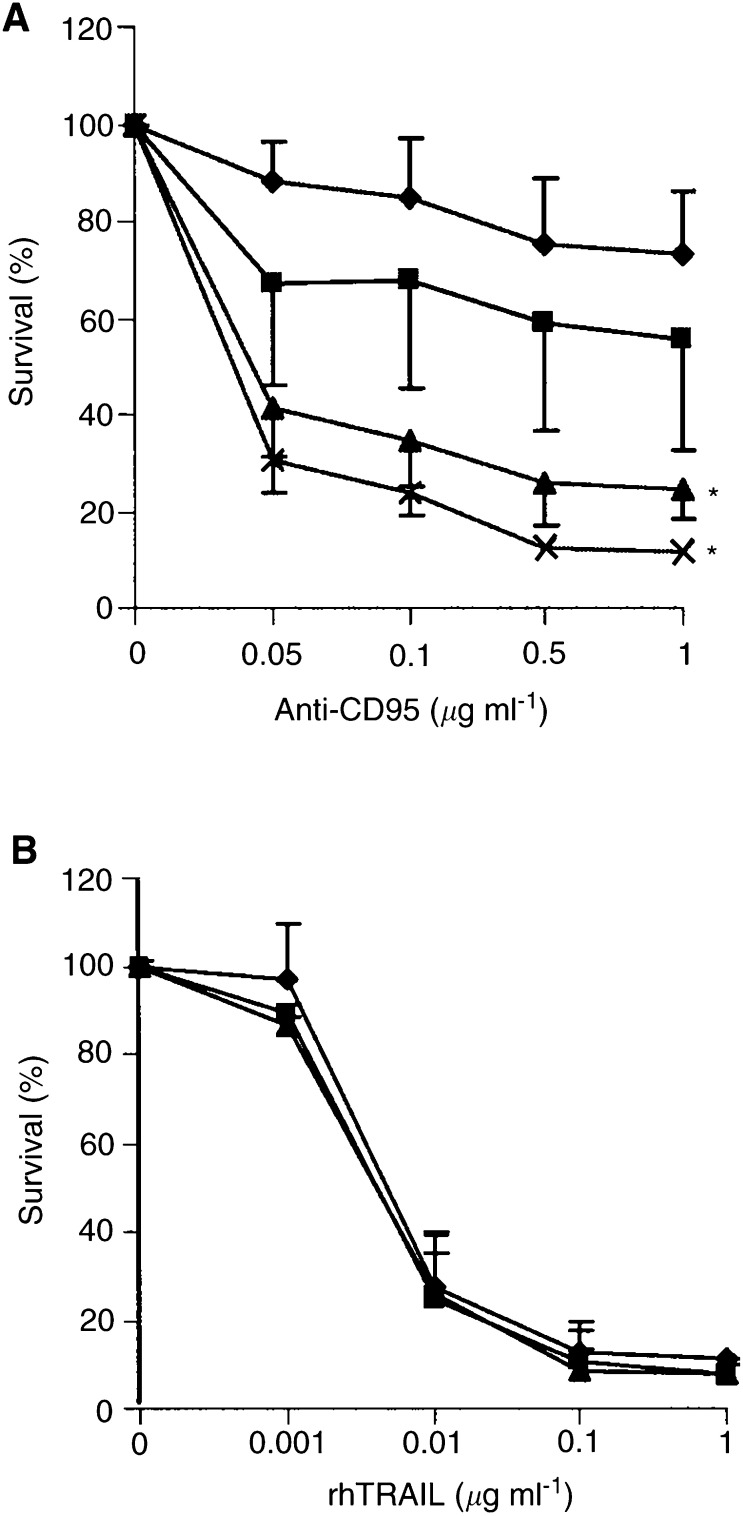
(**A**) Survival of SW948 to anti-CD95 following continuous incubation without (closed diamond) and with 1 U ml^−1^ (closed square), 10 U ml^−1^ (closed triangle) and 1000 U ml^−1^ (asterisk) interferon-*γ* measured by cytotoxicity assays. Values are mean±s.d. of at least three independent experiments. ^*^: survival of SW948 treated with 10 or 1000 U ml^−1^ interferon-*γ* differ from the untreated cells after exposure to anti-CD95 at the concentration of 0.05–1 *μ*g ml^−1^ (*P*<0.05). (**B**) Survival of SW948 to rhTRAIL after continuous incubation without (closed diamonds), and with 10 U ml^−1^ (closed square) and 1000 U ml^−1^ (closed triangle) interferon-γ measured by cytotoxicity assays. Values are mean±s.d. of at least three independent experiments.

The effect of these modulators on rhTRAIL sensitivity was studied with the cytotoxicity assay. Treatment of 5-FU, interferon-*γ* or cisplatin did not show a clear synergistic effect with rhTRAIL sensitivity in any of these cell lines (Figure 13

B and Table 1

**Table 1:** Overview of IC_50_ values for Caco-2, Colo-320 and SW948 for rhTRAIL, anti-CD95, cisplatin (CDDP), 5-FU and combinations of these chemotherapeutic drugs with anti-CD95 or rhTRAIL

	**IC_50_**		
**Conditions**	**Caco-2**	**Colo-320**	**SW948**
TRAIL (*μ*g ml^−1^)	>1.0	>1.0	0.007±0.001
anti-CD95 (*μ*g ml^−1^)	>1.0	>1.0	>1.0
CDDP (*μ*M)	8.9±0.76	2.3±0.45	4.3±0.92
anti-CD95+CDDP (*μ*M)	6.4±1.2	3.8±1.8	3.6±0.42
TRAIL+CDDP (*μ*M)	11.3±3.2	2.5±0.26	4.4±0.20
5-FU (*μ*M)	3.0±0.74	10.6±6.67	3.0±0.46
anti-CD95+5-FU (*μ*M)	13.0±5.0	20.0±2.2	4.0±1.95
TRAIL+5-FU (*μ*M)	2.3±0.6	7.5±2.1	2.75±0.25
1000 U ml^−1^IFN+TRAIL (*μ*g ml^−1^)	>1.0	>1.0	0.006±0.001
1000 U ml^−1^+anti-CD95 (*μ*g ml^−1^)	>1.0	>1.0	0.024±0.008

).

### Comparing TRAIL-receptor modulation with CD95-receptor modulation

**Figure 11:**
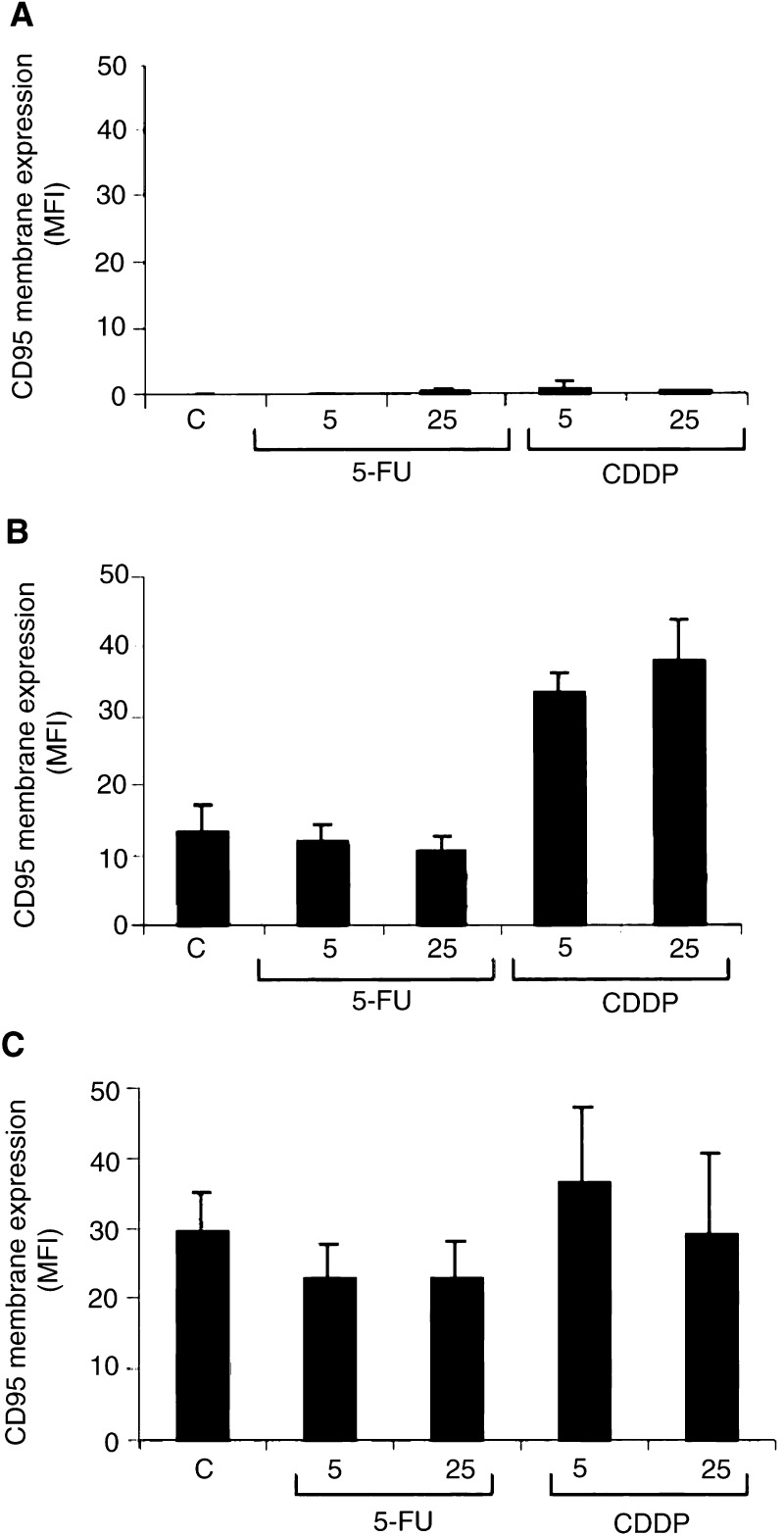
CD95-receptor membrane expression in (**A**) Caco-2, (**B**) Colo320 and (**C**) SW948 in control cells, after 24 h exposure to 5 or 25 *μ*M 5-fluorouracil (5-FU), 5 or 25 *μ*M cisplatin (CDDP) or 100 U ml^−1^ interferon-*γ* (IFN). Membrane expression is determined by flow cytometry and is expressed as MFI. Exposure to 5-FU, cisplatin or interferon-*γ* did not change the percentage at receptor positive cells. Values are mean±s.d. of at least three independent experiments.

**Figure 12:**
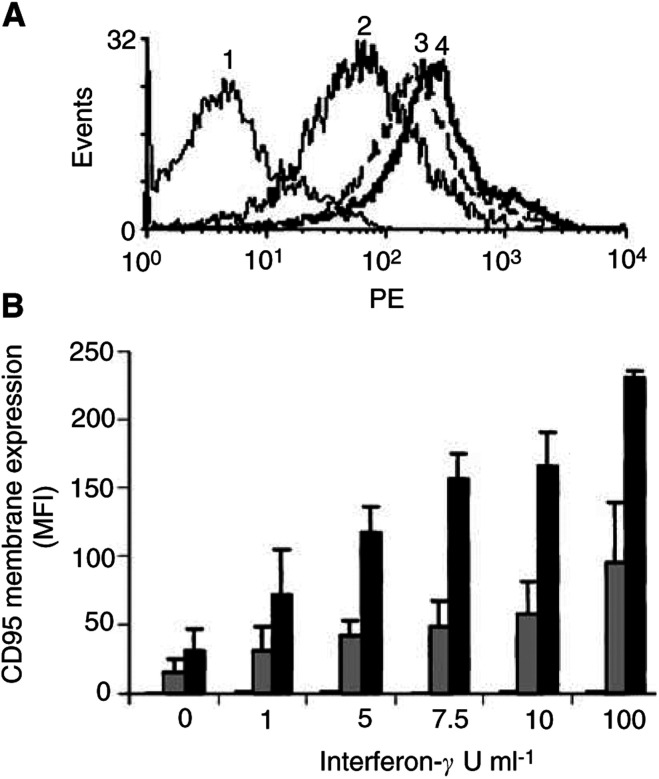
CD95 membrane expression in Caco-2, Colo320 and SW948 after exposure to interferon-*γ*. (**A**) A representative example of increased CD95 membrane expression in SW948 after exposure to interferon-*γ*. Increased CD95 expression was detected as an increased fluorescence intensity of the whole cell population and resulted in a peak shift to the right. (**1**=negative control; **2**=0 U ml^−1^ interferon-*γ*; **3**=10 U ml^−1^ interferon-*γ*; **4**=100 U ml^−1^ interferon-*γ*) (**B**) CD95 membrane expression in Caco-2 (white bars), Colo320 (grey bars) and SW948 (black bars) after 24 h incubation with interferon-*γ*. CD95 membrane expression is determined by flow cytometry and is expressed as MFI. Exposure to interferon-*γ* did not change the percentage of receptor positive cells. Values are mean±s.d. of at least three independent experiments.

The TRAIL-mediated and the CD95-mediated cellular apoptotic pathways share some characteristics. To analyse whether these pathways differ in upregulating their membrane receptors, we studied the effect of 5-FU, cisplatin and interferon-*γ* on the CD95 membrane expression of the cell lines. Basic CD95 membrane expression levels are demonstrated in Figure 5. CD95 membrane expression in Colo320 was slightly lower than in SW948. Caco-2 expressed no CD95 receptor on the membrane. Treatment with 5-FU slightly decreased CD95 membrane expression in Colo320 and SW948 (Figure 11

). Cisplatin increased CD95 surface levels only in Colo320 (∼2 ×) (Figure 11), while interferon-*γ* raised CD95 membrane expression in Colo320 and SW948 (6 × and 8 ×, respectively) (Figure 12

).

**Figure 14:**
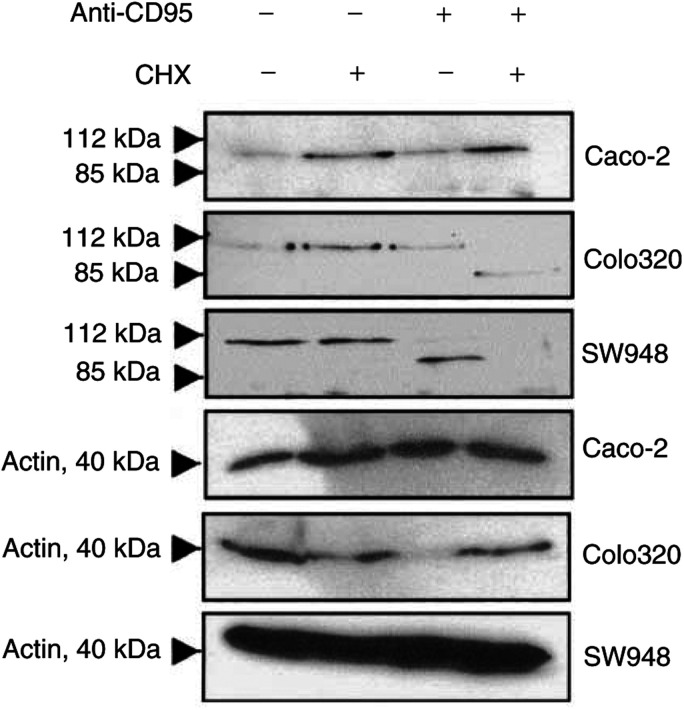
Western blot analysis of PARP-cleavage in Caco-2, Colo320 and SW948 after exposure to cycloheximide (CHX), anti-CD95 or cycloheximide in combination with anti-CD95. Cells were 24 h preincubated with 100 U ml^−1^ interferon-*γ* before cycloheximide and anti-CD95 were added. The full-length PARP (112 kDa) is cleaved in an 85 kDa fragment.

Cytotoxicity experiments showed that none of the cell lines were sensitive to anti-CD95 (Figure 1B). Although both cisplatin and interferon-*γ* could modulate CD95 membrane expression in Colo320 and SW948, only SW948 could be sensitised after treatment with interferon-*γ* (Figures 7 and 13). In the presence of cycloheximide and interferon-*γ*, Colo320 and SW948 became sensitised to anti-CD95 but not Caco-2. Whereas all SW948 cells were apoptotic following treatment with cycloheximide, interferon-*γ* (10 U ml^−1^), and anti-CD95, only ∼10% of Colo320 cells were apoptotic following this treatment, a percentage that raised to ∼40% of Colo320 cells at the highest interferon-*γ* concentration used in this study (100 U ml^−1^) (Figure 7). These findings were confirmed with Western blot analysis of PARP cleavage (Figure 14

). Western blot analysis showed that treatment with anti-CD95 alone resulted in a small induction of FLIP-cleaved intermediate product in SW948, but none in Colo320 or Caco-2 (Figure 9). Cycloheximide in combination with anti-CD95 did not result in a complete loss of the full-length FLIP-L in SW948, which may be related to the relatively low percentage of apoptosis as compared to rhTRAIL-induced apoptosis. As expected, no effect of cycloheximide and anti-CD95 on c-FLIP or PARP cleavage was observed in Caco-2. These results suggest that CD95 membrane expression levels are important as is reflected in the higher sensitivity to anti-CD95 of SW948, the cell line with the highest CD95 membrane levels following treatment with interferon-*γ*. Intracellular inhibitors are also affecting sensitivity to anti-CD95 but a minimal expression level of CD95 is required, since the addition of cycloheximide alone to inhibit the synthesis of short-living antiapoptotic proteins can sensitise SW948 to anti-CD95, but cycloheximide as well as upregulation of CD95 membrane expression by interferon-*γ* were needed to sensitise Colo320 to anti-CD95.

## DISCUSSION

Different rhTRAIL sensitivities were observed in the three colon cancer cell lines used in this study. One explanation for this difference in sensitivity is the different membrane expression of the TRAIL receptors. The highly TRAIL-sensitive cell line SW948 expresses more DR5 than DR4 on the membrane, whereas the two relatively resistant lines Caco-2 and Colo320 express DR4 and DR5 at similar levels. Although DcR1 and DcR2 mRNA were detected in these lines, DcR1 and DcR2 are hardly expressed on the cell membrane in any of the lines. Therefore, in these cell lines the ratio of DR4 and DR5 membrane expression might be a more important factor in determining TRAIL sensitivity than the expression of decoy receptors on the membrane. Another explanation for rhTRAIL resistance is a failure of apoptosis signalling due to mutations or deletions in DR4 or DR5. All known TRAIL receptors are localised on human chromosome 8p21-22 (Ashkenazi and Dixit, 1998), which is a hot spot for chromosomal translocations (Mitelman *et al*, 1997). Deletion or mutational inactivation of DR4 and DR5 has been reported in cell lines (Kim *et al*, 2000; Ozoren *et al*, 2000) and several human tumour types (Pai *et al*, 1998; Shin *et al*, 2001). However, in both our intrinsic rhTRAIL-resistant cell lines, apoptosis was observed when the protein synthesis inhibitor cycloheximide was used. This indicates that both lines are still able to bind TRAIL and to mediate a death signal. It is therefore likely that the TRAIL resistance in these lines is due to an intracellular factor inhibiting the death signal.

Our TRAIL-sensitive cell line SW948 is not sensitive for anti-CD95 antibody. This illustrates that CD95 and TRAIL pathways differ, although it is not completely clear at which points. Since many proteins are involved in both pathways, it has been suggested that CD95L and TRAIL engage similar, or at least partly similar, intracellular apoptotic pathways (Velthuis *et al*, 2002). In case of apoptosis, we observed caspase 3 activation and PARP cleavage. However, in the TRAIL- and CD95-resistant cell lines, caspase 3 activation and thus PARP cleavage was prevented by an intracellular inhibiting protein. There are several inhibitors known to act on the CD95 and the TRAIL pathway. One inhibitor of the TRAIL signalling pathway is FLIP, which structurally resembles caspase 8 and can act as an inhibitor of apoptosis when highly expressed in certain cancers. Human melanoma and colon cancer cells, resistant to TRAIL, were noted to have increased FLIP protein levels. A reduction of FLIP levels was associated with an increased sensitivity to TRAIL-mediated cell death (Zhang *et al*, 2000; Hernandez *et al*, 2001). However in another study, no correlation between FLIP levels and TRAIL sensitivity was observed for colon cancer cell lines (Lacour *et al*, 2001). FLIP antisense oligonucleotides and cycloheximide decreased FLIP protein and restored TRAIL sensitivity in human multiple myeloma cells and thyroid cancer cell lines (Mitsiades *et al*, 2002; Poulaki *et al*, 2002). All our cell lines became (more) sensitive to TRAIL in the presence of cycloheximide. This was associated with a decrease in FLIP protein levels (Figure 9). This suggests that FLIP is involved in TRAIL resistance, but that the level of resistance differs per cell line. Xiao *et al* reported that in TRAIL-resistant, but not in TRAIL-sensitive glioma cell lines FLIP was cleaved to produce the p43 fragment after TRAIL exposure (Xiao *et al*, 2002). Interestingly, in our study, the p43 fragment was present in all cell lines following TRAIL exposure, independent of TRAIL sensitivity. TRAIL resistance may also be determined by different ratios of c-FLIP to caspase 8 (Harper *et al*, 2001), but only in Colo320 the ratio FLIP to caspase 8 was high compared to SW948. In contrast to FLIP protein levels, cycloheximide did not affect Bcl-2, Bcl-X_L_ or Bax levels in the cell lines (data not shown), suggesting that these proteins are not as important as FLIP for TRAIL resistance in these cell lines.

It is likely that TRAIL resistance arises from the combination of several factors and not only from the presence of one specific inhibiting protein. We studied whether the protein expression levels of several important proteins involved in apoptosis could explain the difference in TRAIL sensitivity. Key proteins such as DR4, DR5, FADD, caspase 8 and caspase 3 were expressed in all cell lines and the apoptosis inhibitor FLIP was expressed similar in all cell lines. The proteins studied could not clearly reveal a difference in TRAIL sensitivity.

Treatment with cytotoxic drugs can result in an upregulation of CD95 receptor and TRAIL receptors on the tumor cell membrane. The upregulation of CD95 can sensitise tumour cells to the CD95 death signal (Timmer *et al*, 2002). In the present study, we have found that cisplatin treatment increased the CD95 membrane expression. We have investigated whether this can also be an important mechanism in sensitisation of tumour cells to rhTRAIL. The colon carcinoma cell lines were exposed to 5-FU or cisplatin. Chemotherapy is known to be able to augment TRAIL sensitivity and can result in an upregulation of TRAIL receptors at the mRNA and protein levels (Sheikh *et al*, 1998; Keane *et al*, 1999; Chinnaiyan *et al*, 2000; Wu *et al*, 2000; Nimmanapalli *et al*, 2001). Besides the reported upregulation of DR4 membrane expression in erythroleukaemic cells by ionising radiation, little is known about chemotherapy effects on TRAIL-receptor membrane expression. In the colon cancer cell lines 24 h exposure to 5-FU or cisplatin did not affect TRAIL-receptor membrane expression or TRAIL sensitivity. In contrast to us others did observe that 5-FU can sensitise colon cancer cells to TRAIL (Shimoyama *et al*, 2002). This difference might be caused by cell line-specific characteristics. Lacour *et al* (2001) also showed that doxorubicin and cisplatin did not increase membrane DR4 and DR5 expression in colon cancer cell lines, but did sensitise colon cancer cells to TRAIL-mediated apoptosis. This also supports the idea that apart from upregulation of membrane receptor, intracellular factors may be important to become sensitive to TRAIL-mediated apoptosis.

Interferon-*γ* is known to upregulate CD95 membrane expression and renders cells susceptible to CD95-mediated apoptosis (Fellenberg *et al*, 1997; Ossina *et al*, 1997; Koshiji *et al*, 1998; Ruiz-Ruiz *et al*, 2000). Interferon-*γ* raised the CD95 membrane expression levels only in our two colon cancer cell lines that already expressed CD95 at the cell surface. The TRAIL-sensitive cell line became sensitive to anti-CD95 following interferon-*γ* treatment. However, intracellular effects of interferon-*γ* on the sensitivity to anti-CD95 by either an upregulation of caspases or downregulation of Bcl-2 and Bcl-X_L_ cannot be excluded (Fellenberg *et al*, 1997; Ossina *et al*, 1997; Ruiz-Ruiz *et al*, 2000; Varela *et al*, 2001). Interferon-*γ* did not affect TRAIL-receptor membrane expression or TRAIL sensitivity in our cell lines.

The safety of soluble rhTRAIL without a His-tag or a Flag-tag in chimpanzees, its antitumour efficacy alone and its potentiation effect with chemotherapy (Walczak *et al*, 1999) makes rhTRAIL an interesting anticancer agent. The present study demonstrates that chemotherapy (or interferon-*γ*) affects the TRAIL and CD95 pathway differently. Upregulation of DR4 and DR5 on the surface has not been detected, but removal of intracellular blocks can increase TRAIL sensitivity. On the other hand, upregulation of CD95 receptor on the surface has been observed and can increase anti-CD95 sensitivity. In conclusion, our results demonstrate that anticancer agents modulate the CD95 and TRAIL receptors in different ways. Overcoming intracellular inhibiting factors, such as FLIP, is another option to sensitise tumour cells to TRAIL-mediated apoptosis, especially in cells impaired in drug-induced upregulation of TRAIL receptors on the cell surface.

## References

[bib1] Ashkenazi A, Dixit VM (1998) Death receptors: signaling and modulation. Science 281: 1305–1308 9721089 10.1126/science.281.5381.1305

[bib2] Ashkenazi A, Pai RC, Fong S, Leung S, Lawrence DA, Marsters SA, Blackie C, Chang L, McMurtrey AE, Hebert A, DeForge L, Koumenis IL, Lewis D, Harris L, Bussiere J, Koeppen H, Shahrokh Z, Schwall RH (1999) Safety and antitumor activity of recombinant soluble Apo2 ligand. J Clin Invest 104: 155–162 10411544 10.1172/JCI6926 PMC408479

[bib3] Bradford MM (1976) A rapid and sensitive method for the quantitation of microgram quantities of protein utilizing the principle of protein-dye binding. Anal Biochem 72: 248–254 942051 10.1016/0003-2697(76)90527-3

[bib4] Chaudhary PM, Eby AJ, Bookwater A, Murray J, Hood L (1997) Death receptor 5, a new member of the TNFR family, and DR4 induce FADD-dependent apoptosis and activate the NF-κB pathway. Immunity 7: 821–830 9430227 10.1016/s1074-7613(00)80400-8

[bib5] Chinnaiyan AM, Prasad U, Shankar S, Hamstra DA, Shanaiah M, Chenevert TL, Ross BD, Rehemtulla A (2000) Combined effect of tumor necrosis factor-related apoptosis-inducing ligand and ionizing radiation in breast cancer therapy. Proc Natl Acad Sci USA 97: 1754–1759 10677530 10.1073/pnas.030545097 PMC26508

[bib6] Degli-Eposti MA, Smolak PJ, Walczak H, Waugh J, Huang C, DuBose RF, Goodwin RG, Smith CA (1997) Cloning and characterization of TRAIL-R3, a novel member of the emerging TRAIL receptor family. J Exp Med 186: 1165–1170 9314565 10.1084/jem.186.7.1165 PMC2199077

[bib7] Fellenberg J, Mau H, Scheuerpflug C, Ewerbeck V, Debatin KM (1997) Modulation of resistance to anti-APO-1-induced apoptosis in osteosarcoma cells by cytokines. Int J Cancer 72: 536–542 9247301 10.1002/(sici)1097-0215(19970729)72:3<536::aid-ijc25>3.0.co;2-8

[bib8] Fogh J, Wright WC, Loveless JD (1977) Absence of HeLa cell contamination in 169 cell lines derived from human tumors. J Natl Cancer Inst 58: 209–214 833871 10.1093/jnci/58.2.209

[bib9] Gliniak B, Le T (1999) Tumor necrosis factor-related apoptosis-inducing ligand's antitumor activity *in vivo* is enhanced by the chemotherapeutic agent CPT-11. Cancer Res 59: 6153–6158 10626806

[bib10] Griffith TS, Chin WA, Jackson GC, Lynch DH, Kubin MZ (1998) Intracellular regulation of TRAIL-induced apoptosis in human melanoma cells. J Immunol 161: 2833–2840 9743343

[bib11] Harper N, Farrow SN, Kaptein A, Cohen GM, MacFarlane M (2001) Modulation of tumor necrosis factor apoptosis-inducing ligand-induced NF-kappa B activation by inhibition of apical caspases. J Biol Chem 276: 34743–34752 11461927 10.1074/jbc.M105693200

[bib12] Hernandez A, Wang QD, Schwartz SA, Evers BM (2001) Sensitization of human colon cancer cells to TRAIL-mediated apoptosis. J Gastrointest Surg 5: 56–65 11309649 10.1016/s1091-255x(01)80014-7

[bib13] Ilyas M, Straub J, Tomlinson IPM, Bodmer WF (1999) Genetic pathways in colorectal and other cancers. Eur J Cancer 35: 1986–2002 10711241 10.1016/s0959-8049(99)00298-1

[bib14] Keane MM, Ettenberg SA, Nau MM, Russell EK, Lipkowitz S (1999) Chemotherapy augments TRAIL-induced apoptosis in breast cell lines. Cancer Res 59: 734–741 9973225

[bib15] Kim K, Fisher MJ, Xu SQ, El Deiry WS (2000) Molecular determinants of response to TRAIL in killing of normal and cancer cells. Clin Cancer Res 6: 335–346 10690508

[bib16] Koshiji M, Adachi Y, Sogo S, Taketani S, Oyaizu N, Than S, Inaba M, Phawa S, Hioki K, Ikehara S (1998) Apoptosis of colorectal adenocarcinoma (COLO 201) by tumour necrosis factor-alpha (TNF-alpha) and/or interferon-gamma (IFN-gamma), resulting from down-modulation of Bcl-2 expression. Clin Exp Immunol 111: 211–218 9472684 10.1046/j.1365-2249.1998.00460.x PMC1904867

[bib17] Lacour S, Hammann A, Wotawa A, Corcos L, Solary E, Dimanche-Boitrel MT (2001) Anticancer agents sensitize tumor cells to tumor necrosis factor- related apoptosis-inducing ligand-mediated caspase-8 activation and apoptosis. Cancer Res 61: 1645–1651 11245478

[bib18] Leibovitz A, Stinson JC, McCombs III WB, McCoy CE, Mazur KC, Mabry ND (1976) Classification of human colorectal adenocarcinoma cell lines. Cancer Res 36: 4562–4569 1000501

[bib19] MacFarlane M, Ahmad M, Srinivasula SM, Fernandes-Alnemri T, Cohen GM, Alnemri ES (1997) Identification and molecular cloning of two novel receptors for the cytotoxic ligand TRAIL. J Biol Chem 272: 25417–25420 9325248 10.1074/jbc.272.41.25417

[bib20] Marsters SA, Sheridan JP, Pitti RM, Huang A, Skubatch M, Baldwin D, Yuan J, Gurney A, Goddard AD, Godowski P, Ashkenazi A (1997) A novel receptor for Apo2L/TRAIL contains a truncated death domain. Curr Biol 7: 1003–1006 9382840 10.1016/s0960-9822(06)00422-2

[bib21] Mitelman F, Mertens F, Johansson B (1997) A breakpoint map of recurrent chromosomal rearrangements in human neoplasia. Nat Genet 15(Spec no)4: 417–474 9140409 10.1038/ng0497supp-417

[bib22] Mitsiades N, Mitsiades CS, Poulaki V, Anderson KC, Treon SP (2002) Intracellular regulation of tumor necrosis factor-related apoptosis- inducing ligand-induced apoptosis in human multiple myeloma cells. Blood 99: 2162–2171 11877293 10.1182/blood.v99.6.2162

[bib23] Nimmanapalli R, Perkins CL, Orlando M, O'Bryan E, Nguyen D, Bhalla KN (2001) Pretreatment with paclitaxel enhances apo-2 ligand/tumor necrosis factor-related apoptosis-inducing ligand-induced apoptosis of prostate cancer cells by inducing death receptors 4 and 5 protein levels. Cancer Res 61: 759–763 11212279

[bib24] Ossina NK, Cannas A, Powers VC, Fitzpatrick PA, Knight JD, Gilbert JR, Shekhtman EM, Tomei LD, Umansky SR, Kiefer MC (1997) Interferon-gamma modulates a p53-independent apoptotic pathway and apoptosis-related gene expression. J Biol Chem 272: 16351–16357 9195941 10.1074/jbc.272.26.16351

[bib25] Ozoren N, Fisher MJ, Kim K, Liu CX, Genin A, Shifman Y, Dicker DT, Spinner NB, Lisitsyn NA, El Deiry WS (2000) Homozygous deletion of the death receptor DR4 gene in a nasopharyngeal cancer cell line is associated with TRAIL resistance. Int J Oncol 16: 917–925 10762627 10.3892/ijo.16.5.917

[bib26] Pai SI, Wu GS, Ozoren N, Wu L, Jen J, Sidransky D, El Deiry WS (1998) Rare loss-of-function mutation of a death receptor gene in head and neck cancer. Cancer Res 58: 3513–3518 9721851

[bib27] Pan G, Ni J, Wei YF, Yu G, Gentz R, Dixit VM (1997a) An antagonist decoy receptor and a death domain-containing receptor for TRAIL. Science 277: 815–818 9242610 10.1126/science.277.5327.815

[bib28] Pan G, Ni J, Yu G, Wei YF, Dixit VM (1998) TRUNDD, a new member of the TRAIL receptor family that antagonizes TRAIL signalling. FEBS Lett 424: 41–45 9537512 10.1016/s0014-5793(98)00135-5

[bib29] Pan G, O'Rourke K, Chinnaiyan AM, Gentz R, Ebner R, Ni J, Dixit VM (1997b) The receptor for the cytotoxic ligand TRAIL. Science 276: 111–113 9082980 10.1126/science.276.5309.111

[bib30] Pitti RM, Marsters SA, Ruppert S, Donahue CJ, Moore A, Ashkenazi A (1996) Induction of apoptosis by Apo-2 ligand, a new member of the tumor necrosis factor cytokine family. J Biol Chem 271: 12687–12690 8663110 10.1074/jbc.271.22.12687

[bib31] Poulaki V, Mitsiades CS, Kotoula V, Tseleni-Balafouta S, Ashkenazi A, Koutras DA, Mitsiades N (2002) Regulation of Apo2L/tumor necrosis factor-related apoptosis-inducing ligand-induced apoptosis in thyroid carcinoma cells. Am J Pathol 161: 643–654 12163389 10.1016/S0002-9440(10)64220-4 PMC1850734

[bib32] Quinn LA, Moore GE, Morgan RT, Woods LK (1979) Cell lines from human colon carcinoma with unusual cell products, double minutes, and homogeneously staining regions. Cancer Res 39: 4914–4924 498117

[bib33] Rieger J, Ohgaki H, Kleihues P, Weller M (1999) Human astrocytic brain tumors express AP02L/TRAIL. Acta Neuropathol (Berl) 97: 1–4 9930888 10.1007/s004010050948

[bib34] Ruiz-Ruiz C, Munoz-Pinedo C, Lopez-Rivas A (2000) Interferon-gamma treatment elevates caspase-8 expression and sensitizes human breast tumor cells to a death receptor-induced mitochondria-operated apoptotic program. Cancer Res 60: 5673–5680 11059759

[bib35] Schneider P, Bodmer JL, Thome M, Hofmann K, Holler N, Tschopp J (1997) Characterization of two receptors for TRAIL. FEBS Lett 416: 329–334 9373179 10.1016/s0014-5793(97)01231-3

[bib36] Sheikh MS, Burns TF, Huang Y, Wu GS, Amundson S, Brooks KS, Fornace Jr AJ, El Deiry WS (1998) p53-dependent and -independent regulation of the death receptor KILLER/DR5 gene expression in response to genotoxic stress and tumor necrosis factor alpha. Cancer Res 58: 1593–1598 9563466

[bib37] Sheridan JP, Marsters SA, Pitti RM, Gurney A, Skubatch M, Baldwin D, Ramakrishnan L, Gray CL, Baker K, Wood WI, Goddard AD, Godowski P, Ashkenazi A (1997) Control of TRAIL-induced apoptosis by a family of signaling and decoy receptors. Science 277: 818–821 9242611 10.1126/science.277.5327.818

[bib38] Shimoyama S, Mochizuki Y, Kusada O, Kaminishi M (2002) Supra-additive antitumor activity of 5FU with tumor necrosis factor-related apoptosis-inducing ligand on gastric and colon cancers *in vitro*. Int J Oncol 21: 643–648 12168112

[bib39] Shin MS, Kim HS, Lee SH, Park WS, Kim SY, Park JY, Lee JH, Lee SK, Lee SN, Jung SS, Han JY, Kim H, Lee JY, Yoo NJ (2001) Mutations of tumor necrosis factor-related apoptosis-inducing ligand receptor 1 (TRAIL-R1) and receptor 2 (TRAIL-R2) genes in metastatic breast cancers. Cancer Res 61: 4942–4946 11431320

[bib40] Timmer T, de Vries EG, de Jong S (2002) Fas receptor-mediated apoptosis: a clinical application? J Pathol 196: 125–134 11793363 10.1002/path.1028

[bib41] Varela N, Munoz-Pinedo C, Ruiz-Ruiz C, Robledo G, Pedroso M, Lopez-Rivas A (2001) Interferon-gamma sensitizes human myeloid leukemia cells to death receptor-mediated apoptosis by a pleiotropic mechanism. J Biol Chem 276: 17779–17787 11279136 10.1074/jbc.M100815200

[bib42] Velthuis JH, Rouschop KM, De Bont HJ, Mulder GJ, Nagelkerke JF (2002) Distinct intracellular signaling in tumor necrosis factor-related apoptosis-inducing ligand- and CD95 ligand-mediated apoptosis. J Biol Chem 277: 24631–24637 11980895 10.1074/jbc.M111572200

[bib43] Walczak H, Miller RE, Ariail K, Gliniak B, Griffith TS, Kubin M, Chin W, Jones J, Woodward A, Le T, Smith C, Smolak P, Goodwin RG, Rauch CT, Schuh JC, Lynch DH (1999) Tumoricidal activity of tumor necrosis factor-related apoptosis-inducing ligand *in vivo*. Nat Med 5: 157–163 9930862 10.1038/5517

[bib44] Wiley SR, Schooley K, Smolak PJ, Din WS, Huang CP, Nicholl JK, Sutherland GR, Smith TD, Rauch C, Smith CA (1995) Identification and characterization of a new member of the TNF family that induces apoptosis. Immunity 3: 673–682 8777713 10.1016/1074-7613(95)90057-8

[bib45] Wu GS, Kim K, El Deiry WS (2000) KILLER/DR5, a novel DNA-damage inducible death receptor gene, links the p53-tumor suppressor to caspase activation and apoptotic death. Adv Exp Med Biol 465: 143–151 10810622 10.1007/0-306-46817-4_13

[bib46] Xiao C, Yang BF, Asadi N, Beguinot F, Hao C (2002) Tumor necrosis factor-related apoptosis-inducing ligand-induced death-inducing signaling complex and its modulation by c-FLIP and PED/PEA-15 in glioma cells. J Biol Chem 277: 25020–25025 11976344 10.1074/jbc.M202946200

[bib47] Zhang XD, Franco AV, Nguyen T, Gray CP, Hersey P (2000) Differential localization and regulation of death and decoy receptors for TNF-related apoptosis-inducing ligand (TRAIL) in human melanoma cells. J Immunol 164: 3961–3970 10754286 10.4049/jimmunol.164.8.3961

